# Correction

**DOI:** 10.1080/21501203.2025.2461915

**Published:** 2025-02-06

**Article title**: Nutritional contents and antimicrobial activity of the culinary-medicinal mushroom Leccinum scabrum

**Authors**: M.L Gargano., G. Balenzano., G. Venturella., M. M. Cavalluzzi., N. Rotondo., G. Lentini., F. Cirlincione., G. Mirabile., E. Zapora., M. Wołkowycki., L. Pecoraro & V Ferraro

**Journal**: *Mycology*

**DOI**: 10.1080/21501203.2024.2342519

This article was previously with an error in table 7. This has now been added and republished in the original article.

**Table 7.** Growth inhibition percentages obtained with microdilution assay used to test antimicrobial activity of Leccinum scabrum extracts on pathogenic bacterial strains.

	Bacterial strains							
	*Listeria monocytogenes* ATCC 19114		*Staphylococcus aureus* ATCC 33862		*Salmonella enterica* ATCC 13076		*Escherichia coli* ATCC 25922	
	Growth inhibition		Growth inhibition		Growth inhibition		Growth inhibition	
Conc. (mg/mL)	MAE	UAE	MAE	UAE	MAE	UAE	MAE	UAE
100	+++ 99.80%	+++ 94.90%	+++ 99.75%	+++ 95.38%	+++ 99.76%	+++ 99.79%	+++ 99.83%	+++ 97.97%
50	+++ 92.92%	+++ 79.58%	++ 61.93%	++ 66.43%	+++ 81.77%	+++ 89.94%	+++ 93.40%	+++ 80.07%
25	+++ 74.11%	++ 57.60%	++ 40.82%	++ 46.76%	++ 46.65%	++ 67.62%	+++ 75.51%	++ 66.67%
12.5	++ 39.15%	+ 31.08%	+ 25.04%	+ 20.75%	++ 55.39%	++ 43.44%	++ 51.32%	++ 51.94%
6.25	+ 30.12%	+ 18.33%	+ 24.32%	+ 22.30%	++ 43.22%	++ 42.82%	++ 46.73%	++ 39.26%
3.125	+ 23.97%	+ 15.58%	+ 19.03%	+ 16.33%	++ 37.26%	+ 28.90%	+ 27.61%	+ 19.13%
1.5625	+ 17.50%	+ 12.13%	+ 6.38%	+ 10.63%	+ 18.68%	+ 20.70%	+ 23.18%	+ 20.84%
0.78125	+ 14.19%	+ 11.59%	+ 4.13%	+ 10.84%	+ 5.07%	+ 7.10%	-0.00	+ 10.52%

Antibacterial activity is based on inhibition percentages: low activity (+, ≤35%); moderate activity (++, >35% and ≤70%); high activity (+++, >70%); no antibacterial activity (-). Values (+/-) are referred to the average of three technical replicates.

